# The Role of Family in the Life Satisfaction of Young Adults: An Ecological-Systemic Perspective

**DOI:** 10.3390/ejihpe14100182

**Published:** 2024-10-09

**Authors:** Paula Morales Almeida, Marta Brás, Cristina Nunes, Cátia Martins

**Affiliations:** 1 Departament of Psychology, Sociology and Social Work, University of Las Palmas de Gran Canaria, 35001 Gran Canaria, Spain; 2 Psychology Research Centre (CIP), University of Algarve, Campus of Gambelas, 8005-139 Faro, Portugal; mbras@ualg.pt (M.B.); csnunes@ualg.pt (C.N.); csmartins@ualg.pt (C.M.)

**Keywords:** family, youth, psychological distress, life satisfaction, perceived social support, ecological-systemic

## Abstract

The support provided by parents plays a relevant role in the life satisfaction (LS) of young people, as well as in their level of psychological distress (PD), which are among the important mediators of their well-being. Family structure has also been identified as a protective or risk factor. Hence, the present study provides a more complex analysis of young people’s LS, considering the type of family cohabitation (based on parental presence), the personal conditions of the young people (PS), and their perception of the context (social support; SP). A cross-sectional design was used, and 557 young people, with a mean age of 20.68 (*SD* = 2.23), 50.8% women, 60.7% students, participated. Their levels of psychological distress, perceived social support, life satisfaction, and parental cohabitation were assessed. A mediation and a moderated-mediation model were used. Young people living in a biparental family reveal higher levels of LS and lower levels of PS. A partial mediation was found in the effect of SP on the relation between PS and LS. The moderated-mediation model tested showed that the indirect effects on the paths PS–SP and SP–LS were not significant by the type of parental family cohabitation. Several implications regarding family structure on young people’s LS are discussed.

## 1. Introduction

Youth is an intermediate phase between adolescence and adulthood [1] that entails multiple demands for young people on an individual level, in their interpersonal relationships, and in their social environment [2,3]. Developmental changes, entering higher education and/or the labor market, the need to establish new interpersonal relationships outside the family sphere, and social and economic demands can all pose challenges that interfere with the quality of mental health, leading to psychological distress [4,5]. Its relevance can be due to its impact in later life stages [6,7].

Psychological distress, which can be defined as a state of emotional distress frequently accompanied by symptoms of depression and anxiety [8], can be of particular relevance in adolescence (and affect their future life), since, during this stage, mental health and well-being tend to be more vulnerable [9]. Mental health problems interfere with thoughts, feelings/emotions, behavior, and social relationships [10], can vary in degree of severity, and fulfill the criteria of frequency, intensity, and duration for a mental illness [10]. Mental disorders are the leading cause of non-fatal disease burden among young adults around the world [11]. Several studies have recorded an increase in the prevalence of mental health problems in the last decade, including depression and anxiety, with young adults experiencing these symptoms more frequently than other age groups [12].

Despite the concerning rates of mental illness, most young adults do not have a mental illness and are satisfied with their lives. Life satisfaction, the cognitive dimension of subjective well-being, refers to the level of contentment that is perceived when the individual subject thinks about the various areas of their life or life in general [13]. It can be translated into greater involvement in different life activities and a more positive functioning and attitude towards life (e.g., [6]). In university students, for example, life satisfaction is associated with better academic performance [14], higher levels of well-being, greater perception of social support, better relationships with others, and fewer symptoms of anxiety and stress and other mental health problems [15,16].

Life satisfaction and mental health symptoms depend on the different sources and types of support received by young adults [17,18,19]. A systematic review of the literature [15] found that having sufficient information and material support was associated with fewer mental health symptoms and that having material support and family support was associated with greater life satisfaction. Therefore, family support is among the most important sources of well-being, namely due to their social support [20,21], which protects adolescents’ psychological health, with effects throughout life. Social support involves the extent to which a person perceives and has access to support and resources from their social network [21,22]. There are several studies that have highlighted the role of parental practices and support in several youth and adult psychological health domains [22,23,24]. For example, the parents’ response to positive or negative emotions in childhood has an impact on life satisfaction and psychological distress in young adults. When fathers’ responses are supportive in relation to negative emotions in childhood, they are indirectly associated with less distress in the young adult through a less negative emotional experience in the young adult. Mothers’ supportive responses to negative emotions in childhood were indirectly associated with greater satisfaction with life in the young adult through a greater positive emotional experience in the young adult. Yet, in the case of negative emotions, fathers’ supportive responses in childhood were indirectly associated with less distress in the young adult through a less negative emotional experience in the young adult. Mothers’ supportive responses to negative emotions in childhood, in turn, were indirectly associated with greater satisfaction with life in the young adult through a greater positive emotional experience in the young adult [25].

Perceived social support is one of the protective factors of mental health [6,26,27]. When the possibility of providing support is perceived, that is, that emotional, tangible, or informational help can be given, it promotes a sense of purpose and meaning, which can influence the persons’ well-being [28,29,30]. In the daily scenario, it can arise from several contexts or relations, according to the ties (i.e., number and density) and bonding relations (e.g., family, romantic partner) [31], and, in the case of children and adolescents, three main sources were identified (i.e., family, friends, and school professionals) [23,27]. According to Barrera [28], this identification (i.e., sources and types of support) is very relevant to occur, regardless of its purpose.

Several studies have shown the relation between positive parental support and children’s well-being (e.g., [32,33,34,35]), and, although this can be particularly challenging during adolescence, parents continue to play an important role as a source of support [27,34]. Therefore, there are aspects of family support that can be enhanced or frustrated according to family structure and conditions, which can be considered as predictors or consequences [36]. The triad of psychological distress, social support, and life satisfaction has been recently studied (e.g., [37,38]), and a negative relation between psychological distress and social support was reported, as well as a positive one between this last one and life satisfaction, and its mediation role [37,38].

In the last decade, research has focused on various aspects of family complexity (e.g., [39]) as the features of marital unions (or dissolutions) and their relation to several factors, such as economic and psychological [40], sustaining their relevance for the research on, among others, “intergenerational relations, parent and child development and health and well-being” [40] (p. 35). Also, there was a focus on the differences between cohabitation and marriage [36], and, considering the changes nowadays, the separation of marriage from cohabitation is unclear, and the process of union formation is becoming more diverse, complex, and splintered [40]. Moreover, the overlap of parenthood with several demands of the relation can promote increased distress, and this relation can be more relevant in the case of single parents, which have been associated with higher levels of depression and distress [41]. For example, a Spanish study [42] explored adolescents’ well-being according to different family cohabitation arrangements and found that single-parent families and living in care reveal lower levels of subjective well-being due to the levels of instability (e.g., career, home, area, school). So, the adolescents with fewer changes (i.e., in their case, these were the ones living in a two-parent family) reveal significantly higher well-being. The authors highlight that these results do not mean that living with both parents is the best situation, nor that changes are harmful; there are numerous factors that can influence it (e.g., the previous level of well-being, when the changes occur, level of parents’ caring or conflict).

Furthermore, research has shown that institutionalized young people have a higher risk of mental health problems than their peers living with biological families [43,44]. This increased risk of mental health problems among the institutionalized persists into adulthood. Longitudinal studies have shown that adult mental disorders were significantly higher in adults with a history of institutionalization than in the general population [45]. Therefore, young adults with a history of out-of-home care, when compared to their peers with no care experience, have worse mental health and life satisfaction [15]. Although this is not a new research topic (e.g., [46]), the continuous changes in family forms implies that researchers must constantly examine it [36,40].

Overall, research has supported the relation between family structure and children/adolescents’ well-being (e.g., [33,42,47], indicating that biparental families are a protective factor (e.g., [33,39]. This could be due to more parental adjustment and attachment, with outcomes during childhood and young adulthood (e.g., [33,48,49]).

Moreover, the level of support plays a very important role in children and adolescent life satisfaction and well-being (e.g., [50,51,52]) and reveals an inverse association with depression [27]. Among the various types, family social support has been shown to be associated with lower levels of depression and anxiety, albeit with partial mediation of specific resilience resources [6,53]. Following the Stress Process Model [54], which considered that several types and intensities of stressors that people are subjected to, the level and types of social resources available to them to manage the stressors (which constitute relevant contextual elements), and the ways that it can be exposed are relevant for all processes. So, social support is among the principal mediators in this process and, as an outcome or effect, people’s well-being and life satisfaction are frequently identified.

Given these findings, it is essential to adopt an ecological-systemic perspective, which is based on the work of Bronfenbrenner [55], to understand young people’s life satisfaction in the context of personal, family, and social domains. This perspective emphasizes the importance of viewing individuals within the multiple systems that influence their development (e.g., microsystem, mesosystem, exosystem, macrosystem), recognizing that their well-being is shaped by the interaction between their personal conditions (e.g., psychological distress), family dynamics (e.g., family cohabitation), and broader societal contexts (e.g., social support).

Bronfenbrenner’s ecological systems theory highlights that young adults’ development is influenced by the interaction of various layers of environmental systems. These include the immediate family (microsystem), social and school environments (mesosystem), and larger societal structures (exosystem and macrosystem). This approach helps explain how different types of family structures and social support networks interact to affect the psychological well-being and life satisfaction of young adults.

Thus, while many studies show that psychological distress negatively influences life satisfaction in young adults, it is still unclear whether this relationship is direct or mediated by perceived social support. Moreover, it is important to clarify whether these relationships differ depending on the type of parental family cohabitation.

Hence, the present study provides a more complex analysis of young people’s life satisfaction, with special attention to the type of family cohabitation (based on parental presence), the personal conditions of the young people (psychological distress), and their perception of the context (social support). For this purpose, the objectives are:

1. To describe the characteristics of life satisfaction according to types of parental family cohabitation.
2. To explore the relationship between life satisfaction and personal (psychological distress) and social (social support) situations.
3. To analyze how the social situation (perception of social support) affects the relationship between personal situation (psychological distress) and life satisfaction.
4. To examine the moderating role of the family cohabitation model in the mediating influence of social situation (perception of social support) on the relationship between personal situation (psychological distress) and life satisfaction.

## 2. Methods

### 2.1. Participants

In this cross-sectional, non-probabilistic quantitative study conducted through a survey, a total of 557 young people participated. The participants were selected through snowball sampling. The inclusion criteria required them to be between 16 and 24 years old. It was verified that there were no missing data, the information was within the ranges of each variable, and a multivariate outlier analysis was applied for their elimination (*n* = 43).

Table 1 shows the main characteristics of the study sample (*n* = 514), which was composed mainly of women (50.8%), with an average age of 20.68 years (*SD* = 2.23). The majority were only studying (60.1%), and 48% have or are pursuing university studies. Regarding parental family cohabitation, the majority live in ascending biparental families (51.6%).

G*Power [56] was used to calculate the minimum sample size for this study. For a medium effect size (*f*^2^ = 0.15), with an alpha of 0.05, 11 predictors and a power of 0.95, a sample of 178 participants was required. However, despite the difficulties in calculating sample sizes for complex models with estimation of their effects [57], it is noteworthy that the sample size of this study significantly exceeds plausible estimates.

### 2.2. Measures

#### 2.2.1. Sociodemographic Information

The variables used are presented in Table 1.

#### 2.2.2. Psychological Distress

The DASS-21 Scale for Depression, Anxiety, and Stress was used in its short version [58] (Spanish version: [59]). The scale comprises 21 items across three subdimensions (each with seven items): Depression, Anxiety, and Stress. Items (e.g., “I noticed that I had a dry mouth”, “I found it hard to breathe”, and “I felt afraid without reason”) are rated on a four-point scale: 0 (never), 1 (a little or some of the time), 2 (quite a bit or most of the time), and 3 (very much or almost all the time). Each factor of the scale is scored separately by summing the items that comprise it. There is also a general measure of psychological distress defined by the sum of the 21 items. As the score increases, so does the level of psychological distress. Overall, the total scale demonstrates good internal consistency (α = 0.93) as well as its dimensions (Depression: α = 0.87; Anxiety: α = 0.84; Stress: α = 0.84).

#### 2.2.3. Life Satisfaction

The Satisfaction with Life Scale (SWLS, [60]; Spanish version: [61]) was used. Composed of five items, it assesses the person’s global judgment of life satisfaction. Items (e.g., “In most ways, my life is close to my idea”; “The conditions of my life are excellent”; and “I am satisfied with my life”) are rated on a five-point scale: 1 (strongly disagree), 2 (disagree), 3 (neutral), 4 (agree), and 5 (strongly agree). As the scale score increases, so does the level of life satisfaction. In this study, it exhibits optimal internal consistency (α = 0.84).

#### 2.2.4. Social Support

The Multidimensional Scale of Perceived Social Support (MSPSS) [62] (Spanish version: [63]) was used. This 12-item instrument assesses the perceived level of social support from family, friends, and significant others (each comprising four items). Items (e.g., “I get the emotional support I need from my family”; “There is a special person with whom I can share my troubles and joys”; and “My friends really try to help me”) are rated on a six-point scale: 1 (strongly disagree), 2 (disagree), 3 (slightly disagree), 4 (slightly agree), 5 (agree), and 6 (strongly agree). As the score on the scale increases, so does the level of perceived social support. In this study, the Cronbach’s alpha coefficient for internal consistency was optimal, both for the factors of Family Support (α = 0.93), Friend Support (α = 0.95), and Relevant Person Support (α = 0.87), as well as for the total Perceived Social Support score (α = 0.92).

#### 2.2.5. Parental Family Cohabitation

An ad-hoc, multicategorical, single-choice variable was created to identify the type of family cohabitation of the respondents (adaptation of Dinisman et al. [42]). Participants were asked, “With whom do you live most of the year?”. Fourteen responses were included to comprehensively cover various living arrangements, from living alone to living with extended families. The types of families included (single parent, biparental, reconstituted) with attention to gender and the number of siblings or children. New family units formed through pairing during youth were also included. Finally, based on the study’s interest and statistical conditions regarding the minimum size per characteristic, three categories were distinguished: (1) *No parental family cohabitation* (i.e., participants living alone, sharing an apartment with friends, living with extended family or other arrangements such as support centers, including new family units formed through pairing); (2) *Living in an ascending biparental family* (i.e., participants living with two parents, regardless of sex, cohabitation with siblings, including reconstituted families if applicable); (3) *Living in an ascending single-parent family* (i.e., participants living with one of their parents, regardless of the conditions of arrival into the single-parent family, the sex of the parent or guardian, or the number of siblings).

### 2.3. Procedure

After receiving approval from the Ethics Committee of the University of Las Palmas de Gran Canaria (Reference number CEIH-2024-09), the survey was created online using the Google Forms platform but was only administered in a face-to-face format. Three surveyors were trained to conduct the fieldwork, and an online link was set up to record the information collected by the surveyors in person.

Nine survey locations were randomly selected in the region (Canary Islands, Spain) to distribute the sample characteristics. Guidelines were established to determine the random spaces for the survey (e.g., parks, squares, and sports centers) at different times of the day and on different days of the week.

In the study’s implementation, the surveyors provided information about objectives and requested voluntary participation in the research. Anonymity, confidentiality, and data control were also ensured. Informed consent was then obtained. Responses were collected digitally in a private and controlled database. The questionnaire was administered between 24 January and 2 March 2024, with an average completion time of 15 min.

### 2.4. Data Analysis

Initially the database was cleaned and verified that the values of the variables were within their ranges and that there were no missing values. Subsequently, scores for each participant were calculated for each scale and its factors (psychological distress, life satisfaction, and perceived social support). Multivariate analysis was then used to identify outliers in the three domains. Specifically, outliers were removed using the Mahalanobis distance, Cook’s distance, and leverage [64]. Values were identified as outliers if Mahalanobis distances exceeded the critical value for eleven predictors, with a probability of 0.001 of 13.82 (*n* = 7). Cook’s distance criterion indicated outliers with values greater than Di = 0.0078 (*n* = 42). Leverage was considered for cases with values greater than twice the number of predictors plus two according to the sample size, *h*_11_ = 0.0011 (*n* = 71). Ultimately, two of the three criteria for outlier identification were applied (*n* = 43). The assumptions for the general linear analysis were also checked, and no issues of normality, multicollinearity, homoscedasticity, or independence of errors were detected [65].

Descriptive statistical analyses were used to define the characteristics of the sample and the instrument (e.g., mean, standard-deviation, percentages, frequencies), and bivariate statistical analysis was employed to examine the relationships between the instruments using Pearson’s correlation coefficient, indicating the strength of the relationship according to Cohen [66]. Scores between 0.10 and 0.39 were considered weak, between 0.40 and 0.69 as moderate, between 0.70 and 0.89 as strong, and higher than 0.90 as very strong [67].

**Figure 1:**
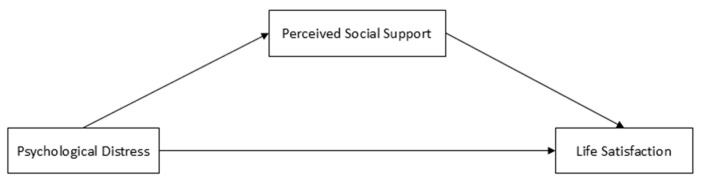
Model 4 (Hayes [68]).

**Figure 2:**
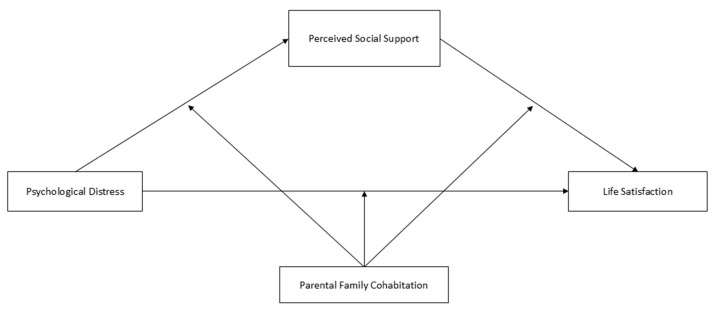
Model 59 (Hayes [68]).

The analysis plan also included examining simple mediation and moderated mediation (Models 4 and 59) using the PROCESS macro 3.5 for SPSS ([68]; Figure 1 and Figure 2). First, Model 4 analyzed the mediation of perceived social support in the relationship between psychological distress and life satisfaction. Second, moderation of the parental family cohabitation type was included in the previous model (Model 59), both in path *a* (between psychological distress and social support) as well as in path *b* (between social support and life satisfaction) and the direct effect path (between psychological distress and life satisfaction). Since the type of parental family cohabitation is a multicategorical variable with three options, the moderation hypothesis test was performed using two dummy-coded variables (*k* − 1). Thus, the product of each dummy variable with each impact variable was calculated to assess the presence or absence of interaction. The models were tested using the Bootstrap method with bias correction at *n* = 5000 and 95% confidence intervals (CIs); a parameter is considered statistically significant if a 95% CI does not include zero.

## 3. Results

### 3.1. Descriptive and Correlational Analysis of the Instruments and Their Relationship with the Type of Parental Family Cohabitation

According to the type of parental family cohabitation (Table 2), the results show that young individuals living in biparental contexts report higher levels of life satisfaction (*M* = 18.57; *SD* = 4.08) and lower of psychological distress (*M* = 16.50; *SD* = 11.90). Participants living in single-parental contexts show the highest level of perceived social support (*M* = 60.09; *SD* = 11.34) and lowest of life satisfaction (*M* = 17.74; *SD* = 4.40).

Regarding correlations (Table 2), life satisfaction was positively and strongly associated with perceived social support (*r* = 0.50, *p* < 0.001), whereas the relationship between psychological distress and perceived social support was negative and weak (*r* = −0.27, *p* < 0.001) and moderate with life satisfaction (*r* = −0.35, *p* < 0.001).

### 3.2. Social Support as a Mediator in the Relationship between Psychological Distress and Life Satisfaction

**Figure 3:**
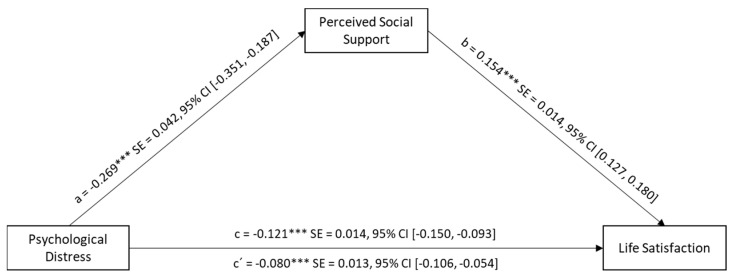
Simple mediation model of social support in the relationship between psychological distress and life satisfaction (*n* = 514). *** *p* < 0.001.

The simple mediation model (Model 4; [68]) used to analyze the effect of perceived social support on the relationship between psychological distress and life satisfaction (Figure 3) suggests that psychological distress is a significant negative predictor of perceived social support (*β* = −0.27, *t* [512] = −6.46, *p* < 0.001, 95% CI = [−0.35, −0.19]). Conversely, social support was a significant positive predictor of life satisfaction (*β* = 0.15, *t* [511] = 11.23, *p* < 0.001, 95% CI = [0.13, 0.18]). However, the direct effect of psychological distress on life satisfaction was also significant (*β* = −0.08, *t* [511] = −5.97, *p* < 0.001). This indicates a partial mediation, with psychological distress remaining a significant predictor of life satisfaction when controlling for social support as a mediator. The total effect (*R*^2^ = 0.12, *F* [1512] = 71.29, *p* < 0.001) identifies a significant model that explains slightly more than 12% of the variance in life satisfaction (*β* = −0.12, *t* [512] = −8.44, *p* < 0.001, 95% CI = [−0.150, −0.093]).

### 3.3. Parental Family Cohabitation as a Moderator in the Mediated Relationship of Perceived Social Support between Psychological Distress and Life Satisfaction

The moderated-mediation model (Model 59; [68]) was employed to define the role of the type of parental family cohabitation as a moderator in the previously tested mediation model. This moderated-mediation revealed that the indirect effects on path a (psychological distress and perceived social support) and path *b* (perceived social support and life satisfaction) were not significant concerning the moderation by the type of parental family cohabitation. However, a significant interaction was found on path *c* (psychological distress and life satisfaction). Therefore, the analysis proceeded with Model 5 [68] based on the findings (Table 3).

With the inclusion of the moderating variable of the type of family cohabitation, the indirect effects and the direct effect of the previous simple mediation model remain significant (no parental cohabitation: *β* = −0.11, *t* = −4.47, *p* < 0.001, 95% CI = [−0.16, −0.06]; living in an ascending biparental family: *β* = −0.03, *t* = −1.64, *p* = 0.101, 95% CI = [−0.07, −0.01]; living in an ascendent single-parent family: *β* = −0.13, *t* = −5.05, *p* < 0.001, 95% CI = [−0.19, −0.08]). First, higher levels of psychological distress are associated with lower levels of perceived social support. Second, higher levels of perceived social support are associated with higher levels of life satisfaction. Third, higher levels of psychological distress correspond to lower levels of life satisfaction.

Regarding moderation (Table 3), the categories of the type of family cohabitation do not predict life satisfaction (no parental cohabitation vs. ascending biparental: *β* = −0.59, *t* = −0.92, *p* =0.361, 95% CI = [−1.85, −0.68]; no parental cohabitation vs. ascending single-parent: *β* = 0.47, *t* = 0.59, *p* = 0.558, 95% CI = [−1.11, 2.05]). However, a significant interaction effect of family cohabitation type in the relationship between psychological distress and life satisfaction was identified (*β* = 0.08, *t* (507) = 3.63, *p* = 0.009, 95% CI = [0.02, 0.14]). The moderating effect of parental family cohabitation was significant for young people not living in parental contexts (*β* = −0.111, *p* < 0.001, 95% CI = [−0.160, −0.062]) and for those living in ascending monoparental contexts (*β* = −0.133, *p* < 0.001, 95% CI = [−0.185, −0.081]) but not for those living in ascending biparental contexts (*β* = −0.031, *p* < 0.101, 95% CI = [−0.067, 0.006]). The predictors explained 32% of the variance in life satisfaction, with the interaction contributing 1.7%.

**Figure 4:**
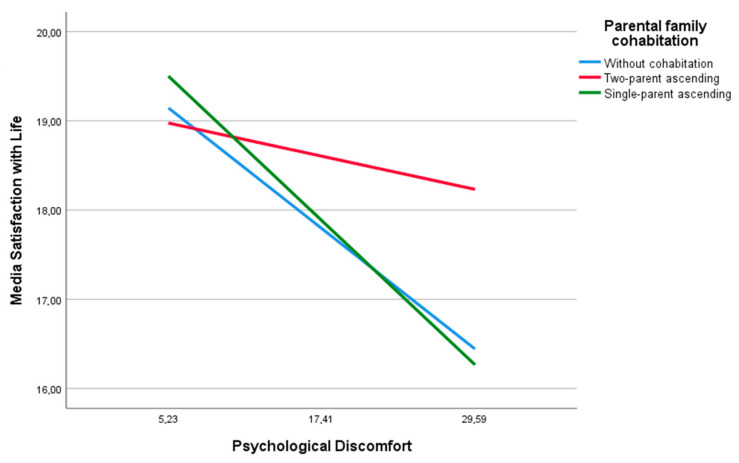
Moderating influence of parent family cohabitation on the association of psychological distress and life satisfaction.

Figure 4 illustrates this moderated relationship, showing how an increase in psychological distress leads to a slight decrease in life satisfaction among youths living in ascending biparental families. This relationship is more pronounced among those without parental living arrangements and those in ascending single-parent systems, with the latter group exhibiting a steeper slope.

In summary, Model 59 demonstrated that psychological distress has a greater negative effect on life satisfaction among young people living in non-parental or single-parent families, while this effect is weaker for those living in biparental families.

## 4. Discussion

The aim of the present study was, in line with the Stress Process Model [54], to analyze the effect of the personal conditions of young people (psychological distress), and their perception of the context (social support) on the life satisfaction of young people, considering the type of family cohabitation (based on parental presence). Generally, the total and by-group scores showed that the psychological distress was at a middle level, the perceived social support was high-moderated, and participants were slightly satisfied. Considering the features and descriptive statistics reported, the participants showed a homogeneity in some sociodemographic features (e.g., sex, age, education level, and status) but also a certain heterogeneity in their psychological characteristics assessed (i.e., due to the standard-deviation), revealing a global profile of median sources of stress, anxiety, and even depression, with a not high level of life satisfaction but with a relevant level of social support.

Regarding the first objective, the results reveal slight differences between the type of family cohabitation and the levels of psychological distress, perceived social support, and life satisfaction, where the group that lived with both their parents showed higher levels of life satisfaction and lower levels of psychological distress and social support. Conversely, the group that lived in a single-parent context reported higher levels of psychological distress and social support, and lower levels of life satisfaction. These results are somewhat in line with the literature that shows that, when living with both parents, children reported higher levels of life satisfaction and well-being than in other living arrangements (e.g., [33,34,39,42,69]. Studies also show that single parents struggle more with several risk conditions (e.g., economic, relationship quality, health), which can be reflected in their children’s well-being [36,42,69,70], as can be justified by the family stress model [71]. Yet, the reported social support levels in single-parent groups are interesting and could be due to the fact that families with this structure reveal a close relationship between parents and child, which could be reflected in parents that focus more on their parental role and are more alert to their sons/daughters’ needs (e.g., [34]).

Another relevant result was the levels reported by the single-parents participants’ group, in comparison to no parental family cohabitation. Considering that this last category could include several conditions (e.g., participants living alone, sharing an apartment with friends, living with extended family, or other arrangements such as support centers, including new family units formed through pairing), it is interesting but somewhat difficult to discuss, since this is due to a variety of situations. Studies with children and adolescents living in single-parent families and in foster care found differences in their interpersonal relationships and health due to, for example, changes and the value of stability (e.g., [42]). Overall, in this respect, several factors are identified, such as the level and quality of parents’ communication [42,72], level of conflict [42], family socioeconomic characteristics [42], feelings of family belonging [34], and number of family members [39].

In respect to the second objective, the results reveal that all factors were related, yet stronger and positive associations were found between perceived social support and life satisfaction. The literature that identifies this relation is abundant (e.g., [20,21,70]), and the association between lower levels of psychological distress and higher levels of social support is also varied [70]. Although a negative relation could be expected, the results showed a weaker but positive and significant relation, which could express a non-linear or unstable relation or the influence of other factors not considered in the analysis [65]. Considering the factors related to both variables (e.g., individual, contextual, situational), more research is needed.

Regarding the third objective, which consisted of analyzing how the social situation (perception of social support) affects the relationship between personal situation (psychological distress) and life satisfaction, a mediation model (Model 4; [68]) was tested, and the results showed a partial mediation. So, in the presence of the mediator (social support), the negative relation between psychological distress and life satisfaction remains significant. These results show that, although social support can mediate this relation, which has been reported by recent research [37,38], it does not fully explain. So, although participants with a higher perception of social support could be less vulnerable to psychological stress (i.e., stress, anxiety) and therefore experience more satisfaction in their lives [15,38], the impact of psychological distress on life satisfaction and well-being is still relevant. This could be due to several other stressors and negative life events (e.g., financial concerns, personality, conflicts, changes, instability, health problems) [73,74,75] that intensify the relationship and do not function as a buffer.

Finally, to examine the moderating role of the family cohabitation model in the mediating influence of social situation on the relationship between personal situation (psychological distress) and life satisfaction (objective four), new models were tested according to Hayes (Model 59; [68]). Although the results disclose that the indirect paths of the moderated mediation were not significant concerning the moderation by the type of parental family cohabitation, a significant interaction was found in the relation between psychological distress and life satisfaction. Hayes’ Model 5 was used to test this relation, and the results showed that family living arrangement only relates to personal conditions (distress) and life satisfaction. The literature has exposed that there are circumstances that families suffer (e.g., changes, instability, financial concerns) that affect children/adolescents’ well-being [33,42,47,76], acting as stress and risk factors [36,54].

By including the family structure in the analysis, the explained variance improved compared to Model 4, highlighting the importance of an ecological-systems perspective and the role of family. There are several family-related factors that can affect life satisfaction positively as social support [20,21] and negatively as stress [77,78] and financial concerns (e.g., [79]). Also, this interaction occurs with lower levels of life satisfaction, starting from psychological distress, among youth in non-familial contexts and those in single-parent families. There are several situations that could underlie these results (e.g., pos-COVID-19 financial situation), although Spanish adolescents/young people are among the ones with higher rates of life satisfaction [78,80].

Overall, the inclusion of the family structure in the analysis of the relation between psychological distress, perceived social support, and life satisfaction shows the importance of family features that influence young people’s well-being. Our results showed that biparental families can serve as stressors and buffers of young life satisfaction, and that single-parent or no family cohabitation conditions can have similar effects on young people, that is, young people could experience high levels of life satisfaction. Yet, if liabilities are present (e.g., higher rates of psychological distress), they are more vulnerable and affected.

Several practical implications can be identified, such as the development and implementation of intervention programs targeting youths living in various family contexts to enhance their well-being, according to their features and specificities. Another is the improvement of the functions of educational, social, and healthcare settings in supporting youths, particularly in paying attention to young people living in single-parent families, addressing the relationship between personal conditions and family dynamics to improve life satisfaction, and the promotion of social support mechanisms to enhance life satisfaction.

Underlying the role that family complexity can have in life satisfaction and well-being [40], other factors are important to explore (e.g., financial status or concerns, conflict, changes), as well as other approaches (e.g., a person-focused approach), that would allow for a more refined and enlightening analysis. Another relevant investment could be the increase and diversification of the sample, allowing for the control of relevant covariates. The inclusion of several visions (e.g., parents) could also enrich and deepen the relationship between family studies and youth studies. So, future research could expand ecological-systems studies.

## 5. Conclusions

Considering the complexity and changes that affect families in their daily lives (e.g., [40,78,81], studies have to be diverse and be attentive to several factors. Although the relation between perceived social support and life satisfaction has been studied in a consolidated way, attending to families’ complexity, there is the need to test mediators and moderators. To our knowledge, this study is among the first to emphasize the role of family living arrangements in relation to young people’s subjective well-being, while considering personal mental health conditions and contextual influences. However, this study has several limitations. Firstly, it was conducted within a specific community in an outermost region, namely the Canary Islands, making it necessary to carry out similar studies in other autonomous communities in Spain and in other countries to determine if cultural biases exist. Additionally, it would be advisable to have a larger sample with greater family diversity to further expand the range of family types and assess whether any differences exist.

## Figures and Tables

**Table 1:** Participants’ characteristics (*n* = 514).

Variables	Categories	*M* (*SD*)/*f* (%)
Sex	Men	253 (49.2)
	Woman	261 (50.8)
Age		20.64 (2.30)
	17–18 years	122 (23.7)
	19–21 years	194 (37.7)
	22–24 years	198 (38.5)
Educational and employment status	Study	312 (60.7)
	Work	87 (16.9)
	Study and work	103 (20)
	Neither studies nor works	12 (2.3)
Level of education	No education	3 (0.6)
	Primary education or basic general education	7 (1.9)
	Compulsory secondary education or intermediate vocational training cycle	63 (12.3)
	High school diploma or higher vocational training cycle	195 (37.9)
	University studies	246 (47.9)
Parental family cohabitation	No parental family cohabitation	143 (27.8)
	Living in an ascending biparental family	265 (51.6)
	Living in an ascending single-parent family	106 (20.6)

Notes. *M* (Mean); *SD* (Standard-deviation); *f* (frequency); % (Percentage).

**Table 2:** Means, standard deviations, and correlations of study variables separated by type of parental family cohabitation (*n* = 514).

	No Parental Family Cohabitation (*n* = 143)	Living in an Ascending Biparental Family (*n* = 265)	Living in an Ascending Single-Parent Family (*n* = 106)	Total	*r*		
	*M* (*SD*)	*M* (*SD*)	*M* (*SD*)	*M* (*SD*)	1	2	3
1 PD	17.83 (11.95)	16.50 (11.90)	19.10 (13.05)	17.41 (12.18)	-	-	-
2 PSS	59.92 (11.44)	59.23 (12.44)	60.09 (11.34)	59.60 (11.93)	−0.27 ***	-	-
3 LS	17.78 (4.33)	18.57 (4.08)	17.74 (4.40)	18.18 (4.22)	−0.35 ***	0.50 ***	-

Notes. PD (Psychological Distress), LS (Life Satisfaction); PSS (Perceived Social Support); *M* (Mean); *SD* (Standard Deviation); *r* (Correlations); *** *p* < 0.001.

**Table 3:** Moderated mediation of the parental family cohabitation between psychological distress and life satisfaction of the model mediated by perceived social support (*n* = 514).

	Consequents									
Antecedents	M (Perceived Social Support)					Y (Life Satisfaction)				
	β	SE	*t*	*p*	95% CI	Β	SE	*t*	*p*	95% CI
X	−0.27	0.040	−6.46	0.00	−0.35, −0.19	−0.11	0.02	−4.47	0.00	−0.16, −0.06
M						0.16	0.01	11.70	0.00	0.13, 0.18
W_1_						−0.59	0.64	−0.91	0.36	−1.85, 0.67
W_2_						0.47	0.80	0.59	0.56	−1.11, 2.05
X × W_1_						0.08	0.03	2.63	0.01	0.02, 0.14
X × W_2_						−0.02	0.04	−0.61	0.54	−0.09, 0.05
Constant	64.28	0.88	72.72	0.00	62.54, 66.02	10.29	1.02	10.12	0.00	8.29, 12.29
Conditional effects										
No parental family cohabitation						−0.11	0.02	−4.47	0.00	−0.16, −0.06
Living in an ascending biparental family						−0.03	0.02	−1.64	0.10	−0.07, 0.01
Living in an ascending single-parent family						−0.13	0.03	−5.05	0.00	−0.18, −0.08
						Δ^2^ (X × W) − R2 = 0.017, F _(2507)_ = 0.017, *p* = 0.002				
	R^2^ = 0.075					R^2^ = 0.32				
	F _(1512)_ = 41.720, *p* < 0.001					F _(6507)_ = 40.078, *p* < 0.001				

Notes. X (Psychological Distress); M (Mediation); W (Moderation: Parental Family Living Situation); W_1_ (No Living Situation vs. Ascending Biparental) and W_2_ (No Living Situation vs. Ascending Single-Parent); FC = Family Cohabitation; BiPF = Biparental Family; SiPF = Single-parent family; β (Beta score); *t* (statistic test); *p* (significance level); CI (Confidence Interval); Δ^2^ (R Square change); F (Statistic test).

## Data Availability

The data can be made available for consultation upon request from the corresponding author.
